# Triple-Negative Breast Cancer Brain Metastasis: A Comprehensive Review of Epidemiology, Molecular Pathobiology, and Therapeutic Frontiers

**DOI:** 10.3390/cancers18071179

**Published:** 2026-04-07

**Authors:** Hongli Yang, Yang Zhao, Yue Wang, Xiaoyuan Ma, Jinmei Ling, Xianyi Zeng, Zihuang Li, Guixiang Liao

**Affiliations:** 1 Department of Radiation Oncology, Shenzhen People’s Hospital (The Second Clinical Medical College, Jinan University, The First Affiliated Hospital, Southern University of Science and Technology), Shenzhen 518000, China; yang.hongli@szhospital.com (H.Y.); ling.jinmei@szhospital.com (J.L.); 2 China Unicom Digital Intelligence Medical Technology Co., Ltd., Guangzhou 510320, China; zhaoy655@chinaunicom.cn (Y.Z.); wangy2357@chinaunicom.cn (Y.W.); maxy172@chinaunicom.cn (X.M.); zengxy96@chinaunicom.cn (X.Z.)

**Keywords:** triple-negative breast cancer, brain metastases, treatment, prognosis, radiotherapy, immunotherapy

## Abstract

Triple-negative breast cancer (TNBC) is associated with a high risk of brain metastases (BMs). Although systemic therapies have improved extracranial disease control, the central nervous system (CNS) remains less accessible to numerous agents. As a result, this limited drug penetration makes brain metastases (BMs) remain common in TNBC, which are a leading cause of serious symptoms. This review summarizes recent key advances in triple-negative breast cancer brain metastases (TNBC-BMs), including epidemiology, prognostic stratification, biological mechanisms of CNS tropism and treatment resistance, and evolving management strategies. We discuss potential mechanisms of brain colonization, including the FOXC1-CXCR4 axis, ST6GALNAC5-related interactions with the blood–brain barrier (BBB), and the bidirectional crosstalk between metastatic cells and the brain microenvironment, particularly astrocytes and microglia. Furthermore, we evaluate the evolving clinical management, emphasizing the transition from whole-brain radiotherapy (WBRT) toward more selective local approaches such as stereotactic radiotherapy (SRS) and hippocampal sparing techniques. Concurrently, we examine the integration of CNS active systemic therapy across specific molecular subsets. This review systematically distinguishes standard-of-care interventions from investigational strategies, ultimately underscoring critical evidence gaps within the TNBC-BM landscape.

## Simple Summary

Brain metastases are a major clinical problem in triple-negative breast cancer. Although systemic treatments have improved disease control outside the brain, many drugs still have limited activity in the central nervous system. This review summarizes recent progress in this field. We discuss the risk and prognosis of brain metastases, key biological mechanisms that may support brain spread and treatment resistance, and recent changes in clinical management. These include the move from whole-brain radiotherapy to more selective local treatments and the growing use of systemic therapies with intracranial activity in selected patients. We also distinguish established treatments from investigational approaches and highlight areas where evidence remains limited. This review may help clinicians and researchers better understand the current treatment landscape and the main questions that still need further study.

## 1. Introduction

Breast cancer is one of the most prevalent malignancies in women and the leading cause of cancer-related death around the world. Survival for hormone receptor-positive and HER2-positive subtypes has improved markedly, but triple-negative breast cancer (TNBC) continues to pose a clinical challenge and shows a high propensity to seed the central nervous system (CNS). Traditionally, the CNS was considered a pharmacologic “sanctuary” protected by the blood–brain barrier (BBB) and the blood–tumor barrier (BTB). This view is being refined because modern systemic treatments prolong survival. Patients with effective extracranial disease control live long enough now to develop brain metastases (BMs) [1]. Studies show that about 7% of metastatic breast cancer (BC) will develop BMs, and the risk in TNBC is 2–5 times higher than in hormone receptor-positive disease. TNBC cells exhibit aggressive molecular programs, which enable them to breach the BBB and adapt to the brain’s unique microenvironment.

This report integrates data from epidemiology, basic science, and clinical trials to provide a critical perspective on the biology and management of BMs in TNBC. Instead of simply listing available treatment options, we examine why certain interventions succeed or fail by considering the strength of preclinical and clinical evidence and how the brain microenvironment shapes disease progression and therapeutic response. Through this synthesis, we aim to clarify established knowledge, identify areas of uncertainty, and outline future directions for research and clinical care.

## 2. Epidemiology and Prognostic Stratification

### 2.1. Incidence Patterns and Risk Profiles

Compared with other BC subtypes, TNBC appears to carry a relatively high burden of BM. In review-level data, the reported incidence of BMs in TNBC ranges from 25% to 46%, and approximately one-third of patients may eventually develop BMs [2]. However, these estimates should be interpreted with caution because they derive from a heterogeneous study populations and disease settings [2].

Data on BMs at initial presentation are more limited. In a SEER-based analysis, 322 of 55,115 patients with TNBC had BMs at diagnosis, showing that de novo BM is uncommon overall but still clinically important [3]. In an independent institutional cohort used as an extended validation set, seven of 108 patients had synchronous BMs, including two patients with TNBC [4]. These findings further support the possibility of early CNS involvement in a subset of patients with TNBC [3,4].

Several commonly cited risk factors, including younger age, receptor status, larger tumor burden, higher histologic grade, and high Ki-67, are drawn from broader breast cancer BM literature rather than TNBC-specific analyses [2]. Review-level evidence also suggests that BM in TNBC tends to occur earlier in the metastatic course and often accompanies visceral metastases, especially in the lungs or liver [2]. Overall, the current literature more clearly supports a high CNS metastatic burden in TNBC than a well-defined set of TNBC-specific clinical predictors [2,3].

### 2.2. Prognostic Outcomes

Historically, survival after BM in TNBC has been poor. Clinical studies report a median overall survival (OS) for patients with TNBC-BM is approximately 4.4–4.9 months, which is shorter than the ranges from HER2-positive and Luminal subtypes BC [2]. Beyond survival, BM is also described as having a substantial adverse impact on quality of life, and BC BMs have been associated with mortality approaching 80% within the first year of diagnosis [2]. The present reference set supports poor outcomes in TNBC-BM, but it does not fully justify a firm statement that the survival disadvantage is directly attributable to treatment resistance and short tumor doubling time alone. A cautious interpretation is that the poor prognosis of TNBC-BM may reflect both the lack of subtype-matched targeted treatment options and the generally aggressive clinical behavior of TNBC [2].

Given the heterogeneity of outcomes after BM, prognostic stratification remains clinically relevant. The Karnofsky Performance Status (KPS) is described as reliable prognostic tool, and the Disease-Specific Graded Prognostic Assessment (DS-GPA) is presented as a framework for estimating survival in patients with BMs from different primary tumors [2]. It further notes that the modified breast GPA incorporates four crucial parameters, including age, KPS, BC subtype, and number of BMs, and it has been used as a BC-specific prognostic index [2]. Accordingly, it is more precise to describe the DS-GPA/breast GPA as tools for survival stratification rather than to claim that they specifically identify TNBC patients who will benefit from aggressive local intervention [2].

Additional retrospective evidence from a single-center real-world cohort treated with whole-brain radiotherapy (WBRT) supports the prognostic relevance of the KPS and intracranial disease burden, but this study was based on a mixed BC BM population [4]. In that study, the KPS ≤ 70 and >3 BM were associated with worse survival [4].

A TNBC-specific population-based SEER study developed a prognostic nomogram for TNBC-BM patients at initial diagnosis [3]. The model incorporated age, histologic type, number of extracranial metastatic sites, surgery, and chemotherapy, and it showed acceptable discriminatory performance in the training and validation cohorts [4]. Its performance was also examined in an extended cohort of patients who developed BMs later in the disease course, although the discrimination was somewhat lower [4]. Overall, these data support the nomogram as a practical tool for risk stratification in TNBC-BM, particularly in those with synchronous BMs [3].

## 3. The Molecular Biology of CNS Tropism

**Figure 1:**
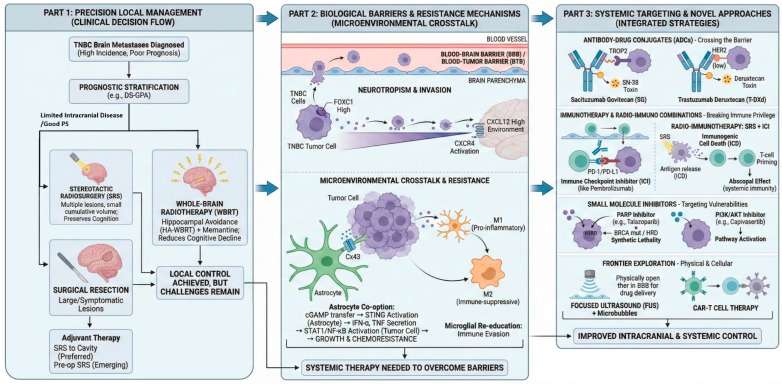
Comprehensive management strategy for TNBC brain metastases: from local control to systemic targeting. DS-GPA: Diagnosis-Specific Graded Prognostic Assessment; PS: performance status; SRS: Stereotactic Radiosurgery; WBRT: whole-brain radiotherapy; HA: hippocampal avoidance; BBB: blood–brain barrier; TME: tumor microenvironment; ADC: antibody–drug conjugate; ICl: immune checkpoint inhibitor; FUS: focused ultrasound; ICD: immunogenic cell death; HRD: homologous recombination deficiency.

In TNBC, BM is not a random event. Rather, it arises through a coordinated set of molecular changes that help cancer cells complete each step of the metastatic process. These steps include the detachment from the primary tumor, survival in circulation, crossing the BBB, and the adaptation to the brain microenvironment (Figure 1).

### 3.1. The FOXC1-CXCR4 Axis: Orchestrating Invasion

The transcription factor forkhead box C1 (FOXC1) has been implicated in TNBC metastasis. In basal-like/TNBC model, FOXC1 overexpression was associated with increased invasion and motility, whereas FOXC1 knockdown had the opposite effect. Mechanistically, FOXC1 facilitates brain homing by directly activating the transcription of CXC chemokine receptor-4 (CXCR4) and pharmacologic inhibition of CXCR4 with AMD3100 attenuated tumor cell migration, invasion, and metastatic dissemination in the zebrafish model [5]. These findings support a functional FOXC1-CXCR4 axis in TNBC metastasis, although the evidence is preclinical and not specific to TNBC-BM.

Nevertheless, this pathway may be relevant to CNS dissemination. In the broader BC literature summarized in [6], CXCL12/CXCR4 signaling is implicated in BM through CXCL12 secretion by astrocytes, high CXCL12/CXCR4 expression in CAFs dervied from BM, and endothelial CXCL12 signaling that may enhance permeability and facilitate migration across the BBB [6]. In addition, TNBC-related stromal CXCL12 signaling has been linked to a protumor microenvironment in broader TNBC models [6]. Taken together, these data support the FOXC1-CXCR4 axis as a biologically plausible contributor to TNBC CNS tropism, while direct TNBC-specific BM evidence remains limited [5,6].

### 3.2. Breaching the Blood–Brain Barrier

Crossing the BBB is a critical step in BM. This process is constrained by tight junctions between endothelial cells and by the surrounding neurovascular unit, including pericytes and astrocytic end-feet [7,8]. In this context, ST6GALNAC5 has been implicated in BC brain tropism and catalyzes the synthesis of the ganglioside GD1α on the tumor cells surface [7]. However, its functional role appears to be model-dependent. In a human BBB in vitro model, ST6GALNAC5 expression decreased the interactions of BC cells with the BBB, challenging the view that it uniformly promotes adhesion or extravasation [7].

In parallel, epithelial-to-mesenchymal transition (EMT) supports metastasis dissemination by facilitating cellular plasticity. EMT has been linked to extracellular matrix (ECM) remodeling, invasion through surrounding tissue, and transendothelial migration as single cells or clusters [8]. Thus, while ST6GALNAC5 may contribute to brain tropism in selected models, its role in BBB transmigration should be interpreted cautiously, and EMT-related plasticity may represent a more general mechanism facilitating extravasation [7,8].

### 3.3. The “Seed and Soil”: Microenvironmental Crosstalk

After entering the brain parenchyma, TNBC cells encounter a distinct and initially restrictive microenvironment. To survive and expand, metastatic cells interact with resident CNS cells, particularly astrocytes and microglia. In the broader BC BM literature, astrocytes may exert antitumor effects, but surviving tumor cells can subsequently exploit astrocyte-mediated signaling to promote growth, chemoresistance, and colonization [8]. Microglia may also display context-dependent functions, with tumor-associated interactions linked to invasion, immune escape, and metastatic outgrowth in brain metastatic models [8].

#### 3.3.1. Astrocyte Co-Option and Gap Junctions

Astrocytes are abundant glial cells in the CNS and can exert both restrictive and tumor-supportive effects in BM. In brain metastatic models, carcinoma cells express PCDH7 to facilitate Cx43-containing gap junctions with astrocytes [9]. These junctions enable tumor cells to transfer the second messenger cGAMP from tumor cells into astrocytes, leading to STING activation and the induction of IFN-α and TNF-α, which in turn activates STAT1 and NF-κB signaling in metastatic cells and promote tumor growth and chemoresistance [9]. Pharmacologic inhibition of gap junctions with meclofenamate or tonabersat suppressed BMs in mouse models, which supports the functional importance of astrocyte tumor coupling in the brain niche [8,9]. However, it is imperative to explicitly acknowledge that astrocyte–tumor interactions are highly multifactorial. The survival and colonization of TNBC cells in the brain are not solely dependent on a single pathway, such as gap junctions, but rather involve a complex, dynamic network that includes metabolic adaptations (e.g., lactate shuttle, glutamine exchange), immune modulation (e.g., cytokine and chemokine signaling), extracellular matrix remodeling, and multifaceted bidirectional crosstalk with various components of the brain microenvironment, including microglia, pericytes, and endothelial cells. Therefore, while gap junction-mediated communication is an important mechanism, it should be viewed as one component of a broader, multifactorial landscape of tumor–stroma interactions in the brain.

#### 3.3.2. Microglial Education and M2 Polarization

Microglia also show context-dependent functions in BM, and available evidence does not support a uniformly tumor-promoting role. In the broader BC BM research, the loss of XIST in BM cells has been linked to exosomal miR-503-mediated microglial reprogramming, with increased PD-L1 expression and suppression of T-cell proliferation, thereby facilitating BMs [8,10]. Additional preclinical evidence from BC BM models showed enrichment of Arg1-positive microglia and increased IL6 and CCL2 secretion after the exposure to BC conditioned medium [11]. In that study, microglia-derived IL6 promoted early colonization through JAK2/STAT3 activation, whereas CCL2 enhanced the recruitment of monocytic myeloid-derived suppressor cells (M-MDSCs), supporting an immunosuppressive microenvironment [11]. VEGF and CXCL2 are described as broader microglia-associated pro-angiogenic mediators rather than TNBC-specific mechanisms [10]. Overall, current evidence supports microglial reprogramming as a potentially important component of brain colonization, but much of the evidence remains preclinical and not TNBC-specific.

## 4. Local Management Strategies: Precision and Preservation

The management of CNS metastasis has shifted away from the historical reliance on WBRT as a universal strategy. Current approaches are highly individualized, with greater emphasis on preserving cognitive function while achieving durable local control.

### 4.1. The Shift from WBRT to Stereotactic Radiosurgery (SRS)

For decades, WBRT was a standard treatment for BM, though its usage usually comes with substantial neurocognitive toxicity. In the prospective JLGK0901 study, 1194 patients with one to 10 newly diagnosed BMs were treated with Gamma Knife alone. Median OS was 10.8 months in both two-to-four and five-to-ten metastasis groups, supporting non-inferior survival in selected patients with five to 10 lesions treated with SRS alone under the tumor burden criteria [12]. A prior retrospective analysis of 1508 patients meeting the JLGK0901 eligibility criteria reached a similar conclusion, supporting the feasibility of SRS alone in the selected patients with up to 10 BMs [13]. These studies could be viewed as broader BM evidence supporting a shift toward SRS in the selected patients.

### 4.2. Postoperative Management: The N107C (Alliance) Trial

Surgical resection remains critical for large symptomatic lesions, but local recurrence rate remains common after surgery alone, necessitating adjuvant intervention. In the N107C (Alliance) Phase III trial, 194 patients with one resected BM and a cavity smaller than 5 cm were assigned to postoperative SRS or WBRT [14]. SRS was associated with longer cognitive deterioration-free survival than WBRT (3.7 vs. 3.0 months; hazard ratio (HR) 0.47) and reduced cognitive deterioration at 6 months (52% vs. 85%), while median OS was similar among the two groups (12.2 vs. 11.6 months; HR 1.07) [14]. Long-term follow-up showed reduced cognitive decline and better quality of life with SRS, whereas intracranial control remained better with WBRT [14,15]. These data support postoperative SRS as a cognitive sparing alternative to WBRT in selected patients, although WBRT provides broader intracranial control [14,15].

### 4.3. Hippocampal Avoidance WBRT (HA-WBRT)

For patients with extensive intracranial disease who are not suitable for SRS, WBRT remains necessary. To reduce neurocognitive toxicity, hippocampal avoidance (HA) techniques have been incorporated into WBRT planning. In the Phase III NRG Oncology CC001 trial, HA-WBRT plus memantine significantly reduced the risk of cognitive failure compared with WBRT and memantine, with adjusted HR of 0.74, while OS, intracranial progression-free survival (PFS), and toxicity were not significantly different between two groups [16]. Longer follow-up also showed better preservation of cognitive function and reduced symptom burden with HA-WBRT and memantine [16]. Hence, these results support HA-WBRT plus memantine as a preferred approach when WBRT is required.

### 4.4. The Debate: Preoperative vs. Postoperative SRS

Preoperative SRS has become a potential option to postoperative SRS for certain resectable BM. A recent systematic review and meta-analysis showed no significant difference between the preoperative and postoperative SRS in survival, local failure, or distant failure. However, the authors reported lower 1-year risks of leptomeningeal disease (LMD) and radiation necrosis with preoperative treatment, without a significant increase in wound complications [17]. A Phase I dose escalation study with an expanded retrospective comparison reported a lower rate of nodular leptomeningeal disease after preoperative SRS than after postoperative SRS (7.4% vs. 27.1%), while local failure and OS were similar [18]. Preoperative SRS may also allow clearer target definition because the intact lesion is treated before cavity change after surgery [17,18]. From these results, preoperative SRS can be viewed as a promising option in selected patients; however, not yet as a definitive replacement for postoperative SRS [17,18].

## 5. Systemic Therapy: The Era of Antibody–Drug Conjugates (ADCs)

Systemic treatment of TNBC with BM remains challenging because CNS drug delivery is limited, and BBB/BTB permeability is heterogeneous. Furthermore, the efficacy of systemic agents is profoundly restricted by active efflux transporters, such as P-glycoprotein (P-gp) and breast cancer resistance protein (BCRP), which actively pump small-molecule drugs out of the CNS. Additionally, stringent molecular size constraints severely limit the penetration of large therapeutic molecules across the uncompromised BBB. These transport mechanisms collectively contribute to the pharmacologic sanctuary of CNS metastases and represent key barriers to effective systemic therapy. In this setting, ADCs have drawn attention because they can deliver potent cytotoxic payloads to tumor cells and have shown intracranial activity in specific BC populations [19,20,21,22].

### 5.1. Sacituzumab Govitecan (SG)

Sacituzumab govitecan (SG) is a TROP2-directed ADC that links an anti-TROP2 antibody to SN-38 through a hydrolyzable linker [19]. In the Phase III ASCENT trial, SG improved PFS and OS over treatment selected by physician’s choice in pretreated metastatic TNBC [19]. However, evidence in TNBC-BM remains limited. In the post hoc ASCENT subgroup analysis, only patients with stable treated BM were included, and SG showed numerically longer median PFS than chemotherapy (2.8 vs. 1.6 months) but similar median OS (7.0 vs. 7.5 months). However, the small sample size limits the firm conclusion [20]. In the French multicenter cohorts (*n* = 99), 31 patients had BM, including active cases. Within the subgroup, median PFS was 3.7 months and median OS was 6.7 months, and partial intracranial responses were observed in evaluable patients treated with SG alone [23]. This suggest that SG may have activity in selected TNBC patients with CNS involvement, yet the evidence is non-randomized and heterogeneous [23].

### 5.2. Trastuzumab Deruxtecan (T-DXd)

T-DXd is not a TNBC directed agent, but it is relevant to the broader ADC discussion because some TNBCs fall into the HER2-low category. However, current evidence is mainly from broader HER2-positive or HER2-low BC cohorts instead of TNBC-specific studies. In the Phase II DEBBRAH trial, T-DXd showed intracranial activity in pretreated HER2-positive patients with stable, untreated, or progressing BMs, with local control of 50% in untreated lesions and 44.4% in progressing lesions [24]. The leptomeningeal cohort included HER2-positive and HER2-low disease. In this group, the median OS was 13.3 months, and the median PFS was 8.9 months. Additionally, 71.4% of these patients achieved prolonged stabilization [25].

In TUXEDO-1, a single-arm Phase II study in HER2-positive active BMs, the intracranial object response rate (ORR) was 73.3% in the intention-to-treat population, with median PFS of 21 months at final analysis [26]. A later multicenter real-world study in patients with active HER2-positive or HER2-low BMs reported intracranial ORR of 65.5% in HER2-positive disease and 66.7% in HER2-low disease [21]. These findings support the CNS activity of T-DXd in HER-positive/HER2-low breast cancer [21,26].

### 5.3. Datopotamab Deruxtecan (Dato-DXd)

Dato-DXd is another TROP2-directed ADC and remains of interest in TNBC because of its target profile. The primary evidence specifically addressing BM comes from the ongoing Phase II DATO-BASE trial [22]. This trial enrolls patients with HER2-negative metastatic BC who have newly diagnosed or progressing BMs alongside a leptomeningeal cohort. Its endpoint is the intracranial ORR assessed by RANO-BM [22]. In addition, the Phase I TROPION-PanTumor-01 study showed promising systemic activity in heavily pretreated metastic TNBC. Specifically, the study reported a confirmed ORR of 31.8%, a median duration of response of 16.8 months, a median PFS of 4.4 months, and a median OS of 13.5 months [27]. In the subgroup of topoisomerase I inhibitor-naïve TNBC, it reported a confirmed ORR of 40% and the median PFS of 7.3 months [27]. The above results mainly support the clinical rationale for further evaluation of Dato-DXd in dedicated BM cohorts, but intracranial efficacy in TNBC-BM has not been established [22,27].

## 6. Immunotherapy and Radio-Immunotherapy Combinations

Among BC subtypes, TNBC is generally considered immunotherapy-relevant. In metastatic TNBC, the clinical role of immune checkpoint inhibitors (ICIs) is established mainly in selected extracranial settings. In contrast, data regarding their efficacy for BMs is still emerging. The current evidence relies largely on Phase II, single-arm, or early combination studies, highlighting a lack of randomized trials specifically dedicated to TNBC-BM [28,29,30,31,32,33].

### 6.1. Immune Checkpoint Inhibition (ICI) in the CNS

The CNS is no longer viewed as completely immune-isolated, providing a rationale for testing ICIs in BM. In a Phase II trial reported by Brastians et al., pembrolizumab (anti-PD-1) was evaluated in active BMs across multiple tissues. The primary endpoint was achieved, with an overall intracranial benefit rate of 42.1%. Among BC patients, the intracranial benefit rate was 37%, and median OS for the whole cohort was 8 months [28,34].

Toxicity also necessitates careful consideration. In the same pembrolizumab study, 52% of patients experienced grade 3 or higher treatment-related adverse events. Notably, two patients developed grade 4 cerebral edema that was considered at least possibly related to the treatment [34]. These findings mainly support future research of ICIs in the CNS disease, but they do not establish pembrolizumab as a TNBC-specific standard for active BM [28,34].

### 6.2. The Abscopal Effect and Radio-Immunotherapy

The combination of SRS with ICI is partly derived from the abscopal effect. Current review-level evidence suggest that the irradiation induces three key biological changes including altering tumor environment, improving antigen presentation, and promoting T-cell activation. These mechanisms provide a clear biological rationale for combining radiotherapy with ICIs [35]. At the same time, the clinical abscopal effect remains uncommon. The current evidence supporting this phenomenon relies primarily on case reports, small case series, and preclinical models, lacking robust prospective validation [35].

Prospective clinical evaluation of radio-immunotherapy in BC BM is ongoing. Trial NCT03449238 is evaluating pembrolizumab plus SRS in patients with metastatic BC and at least two metastases [29]. A Phase II trial NCT03483012 is specifically evaluating atezolizumab with SRS in TNBC-BM. The primary endpoint of this trial is bi-compartmental PFS, while CNS response and safety serve as exploratory endpoints [31]. In addition, a phase Ib study of nivolumab plus SRS in BC BM showed feasibility and no dose-limiting toxicities. However, it was a small, nonrandomized research including mixed subtypes instead of a TNBC-specific efficacy trial [30]. Overall, concurrent radio-immunotherapy is an active investigation area, but it should be carefully described as investigational in TNBC-BM [29,30,31].

### 6.3. Novel Immunotherapeutic Agents

Several novel immunotherapeutic agents are currently under clinical investigation for BMs. Ivonescimab is a bispecific antibody targeting PD-1 and VEGF. Its potential relevance to TNBC-BM is supported by its dual mechanism, as VEGF is involved in both angiogenesis and immune regulation. In a Phase II study in advanced NSCLC, AK112 combined with chemotherapy showed promising antitumor activity and an acceptable safety profile, but it is not TNBC-specific, and intracranial efficacy was not separately reported [32]. Along the same line, other dual targeting immunotherapeutic strategies are being explored to address additional immunosuppressive pathways in the brain microenvironment. Bintrafusp alfa is a bifunctional fusion protein designed to simultaneously target both PD-L1 and TGF-β, the latter being associated with immune suppression in the brain microenvironment. Trial NCT04789668 is an ongoing Phase I/II study in patients with BMs, and the Phase II part includes a BC BM cohort. However, efficacy data are not available [33].

## 7. Targeted Therapies and Pathway Inhibition

Beyond antibody–drug conjugates and immunotherapy, several small molecule strategies are being explored in TNBC. However, the level of evidence is uneven. For PARP and PI3K/AKT pathways inhibitors, most available data come from metastatic TNBC or broader HER2-negative BC populations instead of TNBC-BM trials.

### 7.1. Poly(ADP-Ribose) Polymerase (PARP) Inhibitors

PARP inhibitors are of interest in TNBC because germline BRCA1/2 mutations and homologous recombination defects are enriched in the subtypes. In the Phase III EMBRACA trial, talazoparib improved PFS against chemotherapy in patients with germline BRCA1/2-mutated, HER2-negative advanced BC [36]. A later review further noted that this PFS benefit was reported to be consistent across predefined groups, including patients with a history of BM [37]. However, EMBRACA was not designed as a dedicated study of active BM and did not establish intracranial efficacy [36]. Hence, the available evidence supports PARP inhibition in BRCA-mutated advanced BC, while its specific intracranial activity in TNBC-BM remains insufficiently defined.

Real-world evidence provides supportive but still indirect clinical context. In a retrospective US cohort of patients with germline BRCA-mutated HER2-negative locally advanced or metastatic BC treated with talazoparib, 19% of patients had BM at treatment initiation. The baseline outcomes were broadly consistent with EMBRCA [38]. These results indicate that baseline BM was associated with a higher risk of treatment failure and progression or death, and the study did not report CNS-specific response outcomes. Such findings only prove feasibility in routine practice.

An ongoing Phase I/II trial evaluates SRS with concurrent olaparib followed by durvalumab-based treatment in breast cancer BM [39]. At present, this approach should be regarded as investigational.

### 7.2. PI3K/AKT Pathway Inhibition

Alterations in the PI3K/AKT pathway are frequent in TNBC and are a critical driver of therapeutic resistance. To address this, the randomized Phase II PAKT trial evaluated adding the AKT inhibitor capivasertib to paclitaxel. The combination significantly improved both PFS and OS compared to paclitaxel alone, with the most pronounced clinical benefit observed in patients harboring PIK3CA, AKT1, or PTEN alterations [40]. This study only enrolled metastatic TNBC instead of patients selected for BMs.

The Phase III confirmatory CAPItello-290 trial in TNBC failed to confirm a survival benefit for capivasertib plus paclitaxel in either the overall or biomarker-altered subgroup [41]. Similarly, the BELLE-4 trial of buparlisib in HER2-negative advanced BC failed to show PFS benefits, and it was stopped for futility [42]. Collectively, these findings confirm that PI3K/AKT signaling is biologically relevant in TNBC. However, the clinical benefit of inhibiting this pathway remains unclear, particularly in TNBC-BM.

### 7.3. Etirinotecan Pegol

Etirinotecan pegol (EP), a long-acting irinotecan conjugate, was evaluated in the Phase III ATTAIN trial. This study enrolled metastatic BC patients with previously treated stable BMs. In this study, EP failed to demonstrate superiority over physician’s choice of treatment. It did not improve OS nor did it provide a significant PFS benefit within the CNS [43]. Thus, current evidence does not support EP as a superior option for BC BMs, but it underscores the profound challenge of achieving survival improvements in this patient population.

## 8. Emerging Frontiers: Virus, Cell, and Sound

Several emerging strategies are being explored for TNBC-BM, but the supporting evidence remains limited and uneven. At present, oncolytic virotherapy, CAR-T-cell therapy, and focused ultrasound (FUS) are best regarded as investigational approaches. In most cases, the available data are derived from preclinical studies, early phase trials, or broader BC BM settings rather than TNBC-BM specific studies.

### 8.1. Oncolytic Virotherapy: T-VEC

Talimogene laherparepvec (T-VEC) is a genetically modified herpes simplex virus type 1 designed to induce tumor lysis and stimulate antitumor immunity. In a Phase II study evaluating nonmetastatic TNBC, the combination of T-VEC and neoadjuvant chemotherapy successfully met its primary endpoint. Pathologic evaluation revealed an estimated residual cancer burden (RCB)-0 rate of 45.9%, with 65% of patients achieving an RCB 0–1 response [44].

The phase Ib trial evaluated the combination of T-VEC and atezolizumab in patients with TNBC or colorectal cancer with liver metastases. Notably, the TNBC cohort was limited in size. Among evaluable patients, the combination demonstrated a tolerable safety profile with no dose-limiting toxicities (DLTs) observed. However, clinical efficacy was modest, yielding only a single partial response (PR) [45]. Overall, these findings support a biologic rationale for T-VEC in TNBC, but its clinical value in TNBC-BM remains unproven.

### 8.2. Chimeric Antigen Receptor T-Cell (CAR-T) Therapy

CAR-T therapy for TNBC-BM remains at the preclinical stage. EGFR overexpression is frequently observed in resected TNBC-BM specimens, providing a clear rationale for targeted cellular therapy. EGFR806-targeted CAR-T-cells have also demonstrated reliable antitumor activity both in vitro and in orthotopic human xenograft models. Furthermore, direct intracranial administration of this therapy significantly prolonged survival [46]. These findings support the biological feasibility of this targeting strategy in TNBC-BM models, but they do not yet constitute clinical evidence in patients.

B7-H3 has emerged as a promising therapeutic target supported by clinicopathologic data, demonstrating its widespread expression across BC BMs [47]. However, that descriptive study did not evaluate CAR-T therapy. In a separate preclinical study, dual targeting of B7-H3 and CSPG4 showed antitumor activity in TNBC patient-derived xenograft models [48]. Importantly, these preclinical findings are limited by their reliance on systemic TNBC models, which do not accurately recapitulate the TNBC-BM microenvironment. While CAR-T-cell therapy represents a promising investigational modality, it remains in its nascent stages. Critical translational hurdles, specifically regarding optimal antigen selection and mitigation of on-target/off-tumor toxicities, remain unresolved.

### 8.3. Focused Ultrasound (FUS) for BBB Disruption

Focused ultrasound represents an investigational, noninvasive modality designed to cross the BBB or BTB. For BM specifically, magnetic resonance-guided FUS (MRgFUS) coupled with microbubbles is being evaluated to augment the intracranial penetrance of systemic therapeutics. However, the current evidence remains confined primarily to preclinical models and early-phase clinical trials [49,50].

In an MDA-MB-231Br BC BM model, low-intensity FUS enhanced intratumoral penetrance. Also, the combination of low-intensity FUS with paclitaxel and liposomal doxorubicin significantly delayed intracranial tumor progression and extended median survival [51]. While these preliminary data are promising, they have yet to translate into proven clinical efficacy. Consequently, FUS should be classified as an investigational drug delivery modality instead of a standard-of-care therapeutic option for TNBC-BM.

**Figure 2:**
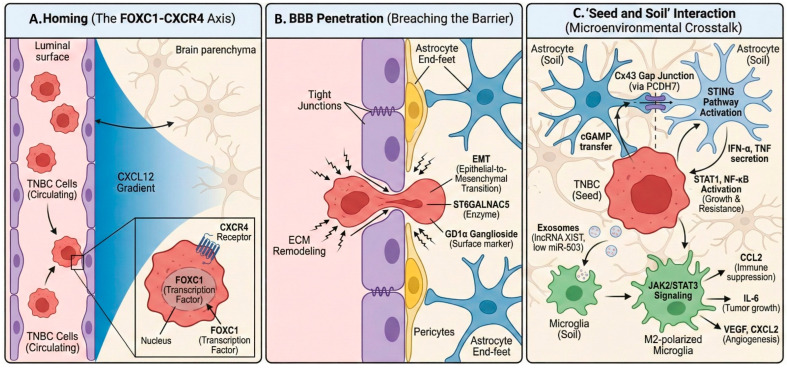
Molecular mechanisms of TNBC central nervous system tropism. (**A**) TNBC cells expressing CXCR4 are attracted to brain-derived CXCL12, promoting adhesion to CNS vasculature. (**B**) TNBC cells undergo EMT and utilize surface molecules like GD1a, aided by enzymes, to disrupt tight junctions and penetrate the BBB. (**C**) TNBC ‘seeds’ establish symbiotic interactions with resident brain ‘soil’ (astrocytes, microglia), hijacking signaling pathways to promote survival, growth, and immune evasion.

A summary of the key therapeutic modalities and their landmark trials discussed in this review is provided in Table 1. Accordingly, FUS remains an investigational drug delivery approach rather than a standard treatment for TNBC-BM (Figure 2).

## 9. Conclusions

TNBC brain metastasis remains a major clinical challenge. Outcomes are poor, and effective intracranial treatment is still limited. Current evidence supports a multifactorial model of CNS spread. Tumor-intrinsic programs, BBB interactions, and brain microenvironmental adaptation all appear to contribute rather than any single dominant mechanism [5,7,9].

Some points are relatively clear. Local therapy remains a core part of management. In the broader brain metastasis literature, radiotherapy paradigms have become more selective, with reduced routine reliance on WBRT and greater use of SRS and HA approaches in selected settings. The goal is to maintain intracranial control while reducing neurocognitive harm [12,15,16]. It is also likely that the increasing clinical detection and relevance of CNS disease in TNBC reflect multiple factors, including longer survival and improved neuroimaging [1,2].

At the same time, several uncertainties remain. Systemic treatment options have expanded, but the quality and relevance of the evidence are not the same across agents and subtypes. Sacituzumab govitecan has shown intracranial activity in TNBC with BMs. Trastuzumab deruxtecan has shown more consistent intracranial activity in prospective studies of HER2-positive BC BMs. These findings should therefore be interpreted in the context of different disease subtypes and study settings [23,24,26]. Similarly, radio-immunotherapy combinations, adoptive cellular therapies, and MR-guided focused ultrasound-based approaches are still early strategies. At present, they should be considered promising but investigational, not established components of TNBC brain metastasis care [29,30,46,49,50].

Several late-phase studies have also reported negative or equivocal results. This supports a cautious interpretation of emerging therapies [41,43]. Further progress will likely require better patient selection, stronger biomarker-linked translational work, and more rigorous intracranial trial design. Clearer distinction between different brain metastasis settings and stronger biomarker-linked translational data will also be needed before these approaches can be defined more confidently in clinical practice.

Overall, progress in TNBC brain metastasis will likely require closer integration of CNS biology, treatment delivery, and intracranial clinical evaluation [1,2,5,7,9].

## Figures and Tables

**Table 1:** Summary of key interventions, landmark trials, and emerging modalities in TNBC brain metastasis.

Agent/Modality	Class	Trial/Study	Key Findings	Refs.
Sacituzumab govitecan	ADC (TROP2)	ASCENT (Phase 3)	Benefit in stable BM; ORR observed in active BMs.	[19]
Trastuzumab deruxtecan	ADC (HER2)	DEBBRAH /TUXEDO-1	High intracranial response in HER2− low and HER2+ BM.	[24,26]
Pembrolizumab	PD-1 inhibitor	Brastianos et al.	37% intracranial benefit in BC cohort.	[28]
Talazoparib	PARP inhibitor	EMBRACA	PFS benefit extends to gBRCAm patients with BM.	[34,37]
SRS vs. WBRT	Radiotherapy	JLGK0901 /N107C	SRS non-inferior survival; WBRT causes cognitive decline.	[12,14]
Preop SRS	Radiotherapy	Meta-analyses	Reduces leptomeningeal disease (LMD) risk vs. postop.	[18]
EGFR806 CAR-T	Cellular therapy	Preclinical	Eradication of intracranial TNBC xenografts.	[17,46]
Focused ultrasound	Device	Preclinical/early clinical	Transient BBB opening enhances drug delivery.	[50,51]

## Data Availability

The original contributions presented in this study are included in the article. Further inquiries can be directed to the corresponding authors.

## Abbreviations

The following abbreviations are used in this manuscript:

TNBC	triple-negative breast cancer
ER	estrogen receptor
PR	progesterone receptor
HER2	human epidermal growth factor receptor 2
CNS	central nervous system
BMs	brain metastases
TNBC-BMs	triple-negative breast cancer brain metastases
BBB	blood–brain barrier
WBRT	whole-brain radiotherapy
SEER	Surveillance, Epidemiology, and End Results
OS	overall survival
DS-GPA	Disease-Specific Graded Prognostic Assessment
FOXC1	forkhead box C1
EMT	epithelial-to-mesenchymal transition
ECM	extracellular matrix
PCDH7	protocadherin 7
Cx43	connexin 43
IFN-α	interferon-α
TNF	tumor necrosis factor
lncRNA	long non-coding RNA
M-MDSCs	monocytic myeloid-derived suppressor cells
SRS	Stereotactic Radiosurgery
HA	hippocampal avoidance
CSF	cerebrospinal fluid
ADCs	antibody–drug conjugates
SG	sacituzumab govitecan
TPC	treating physician
T-DXd	trastuzumab deruxtecan
Dato-DXd	datopotamab deruxtecan
ICI	Immune Checkpoint Inhibition
ICD	immunogenic cell death
PARP	poly (ADP-ribose) polymerase
HRD	homologous recombination deficiency
EP	etirinotecan pegol
T-VEC	talimogene laherparepvec
CAR-T	Chimeric Antigen Receptor T-Cell
FUS	focused ultrasound
MRgFUS	MRI-guided focused ultrasound
ORR	object response rate
PFS	progression-free survival
